# Right atrial strain in atrial fibrillation: the hidden side of the moon

**DOI:** 10.3389/fcvm.2025.1578524

**Published:** 2025-05-30

**Authors:** Eleonora Ruscio, Francesca Augusta Gabrielli, Gaetano Pinnacchio, Francesco Raffaele Spera, Federica Giordano, Roberto Scacciavillani, Maria Lucia Narducci, Gianluigi Bencardino, Francesco Perna, Filippo Crea, Gemma Pelargonio

**Affiliations:** ^1^ Department of Cardiovascular Sciences, Fondazione Policlinico Universitario Agostino Gemelli IRCCS, Rome, Italy; ^2^ Department of Clinical and Molecular Medicine, Sapienza University, Rome, Italy; ^3^ Cardiology Department, Sant'Andrea University Hospital, Rome, Italy; ^4^ Cardiothoracic Department, University Hospital Santa Maria Della Misericordia, Udine, Italy; ^5^ Cardiology Institute, Catholic University of Sacred Heart, Rome, Italy; ^6^ Center of Excellence of Cardiovascular Sciences, Ospedale Isola Tiberina—Gemelli Isola, Rome, Italy

**Keywords:** atrial fibrillation, strain imaging, echocardiography, atrial cardiomyopathy, catheter ablation

## Abstract

**Introduction:**

Left atrial (LA) remodeling in atrial fibrillation (AF) is well studied, whereas right atrial (RA) alterations remain poorly characterized. This study evaluates bi-atrial strain dynamics in patients with AF undergoing catheter ablation (CA).

**Methods:**

A total of 56 consecutive patients undergoing AF ablation were prospectively evaluated using speckle-tracking echocardiography and electrophysiological study before and after CA (median follow-up: 7 ± 3 months). A control group of 32 individuals undergoing CA for paroxysmal atrioventricular nodal reentrant tachycardia, without structural heart disease, was included for comparison.

**Results:**

Compared to controls, AF patients exhibited significantly lower RA strain parameters (right atrial peak strain, reservoir phase, pRASr: 22.1 ± 12.6% vs. 29.8 ± 12.7%, *p* = 0.009) and greater RA mechanical dispersion (defined as the standard deviation of the time-to-peak positive strain, from the three RA segments, corrected for R-R interval, SD-regional-RA-TTP-N: 0.048 ± 0.015 vs. 0.038 ± 0.009, *p* = 0.020). Patients with persistent AF demonstrated a more pronounced RA dysfunction than those with paroxysmal AF (pRASr: 15.9 ± 11.7% vs. 24.8 ± 12.1%, *p* = 0.017; SD-regional-RA-TTP-N: 0.062 ± 0.030 vs. 0.043 ± 0.023, *p* = 0.016), despite comparable LA strain values. RA function correlated with both LA strain and volume parameters, and with the extent of abnormal LA electroanatomical substrate (pRASr and left atrial peak strain, reservoir phase, pLASr: *r* = 0.594, *p* < 0.001; pRASr and low-voltage LA area: *r* = −0.316, *p* = 0.018). Notably, RA parameters, rather than LA indices, were significantly reduced in patients with post-ablation AF recurrence (pRASr: 14.1 ± 11.7% vs. 24.6 ± 13.5%, *p* = 0.039; SD-regional-RA-TTP-N: 0.054 ± 0.031 vs. 0.032 ± 0.010, *p* = 0.002). However, the CHA2DS2-VASc score remained the sole independent predictor of AF recurrence (HR 1.47, 95% CI 1.003–2.158, *p* = 0.048).

**Conclusion:**

RA function, assessed through strain imaging, was progressively impaired in more severe AF subtypes, strongly correlating with bi-atrial mechanical and electroanatomical properties. Furthermore, RA function was associated with AF recurrence after catheter ablation, highlighting its potential prognostic value.

## Graphical Abstract

Right atrial strain in atrial fibrillation: the hidden side of the moon. RA function, assessed through strain imaging, was progressively impaired in more severe AF subtypes, strongly correlating with bi-atrial mechanical and electroanatomical properties. Furthermore, RA function was associated with AF recurrence after catheter ablation, highlighting its potential prognostic value. AF, atrial fibrillation; CA, catheter ablation; SHD, stable heart disease; RA, right atrium; LA, left atrium; pRASr, right atrial peak strain (reservoir phase); pLASr, left atrial peak strain (reservoir phase); PAF, paroxysmal atrial fibrillation; PeAF, persistent atrial fibrillation; SR, sinus rhythm; IQR, interquartile range.

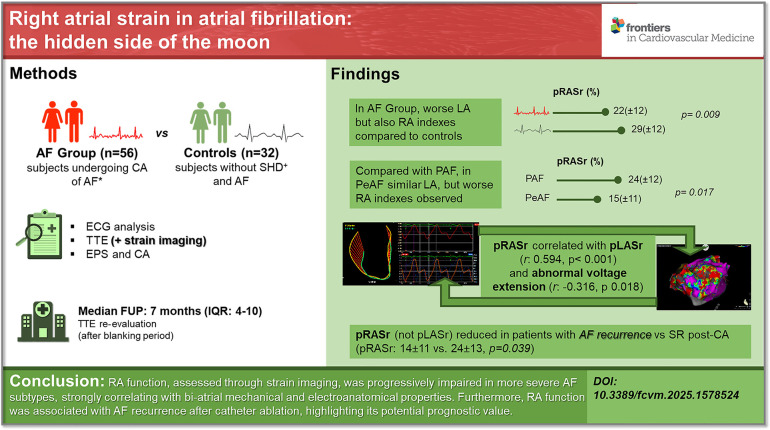

## 1 Introduction

Atrial fibrillation (AF) is the most common sustained arrhythmia, with a progressively increasing “global burden” because of the aging of the population, higher prevalence of risk factors, and better detection strategies (1). AF is associated with left atrial (LA) enlargement, remodeling, and fibrosis, caused by LA pressure and/or volume overload (2).

In this setting, atrial remodeling is a complex process, still poorly understood, that includes atrial fibrosis, fatty infiltration, inflammation, ion channel dysfunction, atrial ischemia, and vascular remodeling (3).

Among AF treatments, catheter ablation (CA) has been demonstrated to be superior compared to antiarrhythmic drugs in terms of maintaining sinus rhythm (SR), improving the quality of life and symptoms of patients with AF; however, long-term recurrences are still a problem (4). Considerable effort has been made to find predictors defining for which patients CA is better suited and the ones which are mostly associated with AF recurrences after CA (5, 6). Although many factors have been related to recurrences, even by combining multiple predictors together, their predictive power always remains modest. Recently, echocardiographic strain imaging has emerged as a useful tool to detect subclinical LA dysfunction (7, 8). Interestingly, impaired LA strain has been reported to predict both AF occurrence in cryptogenic cerebrovascular accidents (9) and recurrence of AF after electrical cardioversion (10) and CA (11), suggesting a profound link between electrical and mechanical function. In contrast, right atrium (RA) remodeling has been less investigated in AF, although the bi-atrial substrate has been described in the more severe subtypes of AF and particularly in patients exhibiting comorbidities (12–16).

As most studies have focused on fibrosis and scar assessment in the LA, structural changes have remained nearly unexplored in the RA, and the relationship between electrical and functional features requires deeper analysis.

The aim of the study was to investigate the role of bi-atrial function, mostly focusing on the right atrium, in patients with AF undergoing CA, to better understand the pathophysiology of AF and bi-atrial cardiomyopathy.

## 2 Methods

### 2.1 Patient population and involvement statement

We enrolled consecutive patients undergoing CA of symptomatic non-valvular AF, according to current guidelines (4, 13). The exclusion criteria were as follows: permanent atrial fibrillation, more than moderate mitral and/or aortic stenosis/regurgitation, prosthetic valves, underlying cardiomyopathies, active malignancy or systemic inflammatory disease, ongoing thyroid disorders, severe psychiatric diseases, drug/alcohol addiction, and suboptimal echocardiographic windows. At the same time, we enrolled a group of subjects who underwent CA of paroxysmal atrioventricular node-reentrant tachycardia, but without structural heart disease (assessed by clinical history, non-invasive cardiac examination, and, when deemed appropriate, by invasive assessment), that served as the control group.

The study protocol was approved by our institutional ethics committee. Written informed consent was obtained from each patient.

We outlined the research topic by sharing with our patients the gap in evidence in the literature. Patient-centered research questions were implemented and translated in the execution phases of the study. We provided basic educational material to facilitate the patients' comprehension and elicit their feedback. Partnering with patients improved the value of this study, while maintaining the scientific rigor of the work.

### 2.2 Electrocardiographic analysis and echocardiographic assessment

A resting 12-lead ECG acquired at a paper speed of 25 mm/s with leads placed in standard position was obtained on admission to assess rhythm, heart rate, and *P*-wave characteristics, among others.

Measurements were conducted using digitized tracings with calipers allowing 1 ms resolution at a screen velocity of 200 mm/s.

*P*-wave analysis was performed pre-CA in patients in SR on admission or post-ablation in patients admitted with AF. The following data were acquired, as described elsewhere (17): the longest and the shortest *P*-wave duration (Pmax and Pmin, respectively; ms), *P*-wave dispersion (Pd, ms), *P*-wave amplitude in lead DII (*P*-wave A, mV), *P*-wave terminal force in lead V1 (PTFV1, mV × ms).

All patients underwent echocardiographic examination before ablation, performed by one experienced cardiologist who was blind to patient characteristics and outcome using commercially available Toshiba Artida (Toshiba Medical Systems, Tokyo, Japan) and Philips EPIQ7 (Philips Medical Systems, Bothell, WA, USA) ultrasound machines, equipped with a 3.5 MHz phased array probe.

Standard echocardiographic parameters were measured according to current international recommendations (18, 19). Both LA volume (LAV) and RA volume (RAV) were indexed for body size (LAVi and RAVi, respectively). LA and RA emptying fractions (LAEF and RAEF, respectively) were calculated as a percentage based on the following formula: {[LA (RA) maximum volume − LA (RA) minimum volume]/LA (RA) maximum volume} × 100%. The measurements were averaged from three consecutive cardiac cycles in the control group and in patients with AF while in SR and from five consecutive cardiac cycles during AF. Datasets were digitally stored and exported to a remote workstation for offline analysis.

After a standard examination, semi-automated two-dimensional (2D) strain imaging was performed. All strain parameters were assessed using speckle-tracking echocardiography (STE) with external commercially available software (TomTec Imaging Arena, Unterschleissheim, Germany) (20).

A dedicated four-chamber view was obtained to quantify LA strain (19). The region of interest (ROI) was selected using the point-and-click method and manually adjusted afterward to outline the atrial wall, extrapolated across pulmonary veins and atrial appendages that were excluded, to ensure the reliability of the automatic calculation. The zero reference point for image analysis was taken at the onset of the QRS complex (R-R gating), corresponding to the ventricular end-diastole. Thus, the first positive peak was analyzed as LA reservoir strain (pLASr, %). Moreover, the corresponding time-to-peak value was calculated (TTP, ms). The image processing algorithm automatically subdivided the atrial wall into three segments distributed in the septum, lateral, and posterior (roof) walls. LA mechanical dispersion was defined as the standard deviation (SD) of time-to-peak positive strain (SD-regional-LA-TTP) from the three LA segments. This parameter was included to quantify the “temporal heterogeneity” in myocardial deformation across different LA segments. To enhance reliability, a correction for R-R interval has been applied (SD-regional-LA-TTP-N). As stated elsewhere, higher values of SD-TPS are correlated with a greater degree of LA dispersion (21).

To determine the RA strain (RAS) parameters, we used an RV-focused apical four-chamber view. Nomenclature equivalent to that of the LA was used [RA reservoir strain, pRASr, %; time-to-peak (TTP), ms]. The RA dispersion parameters were derived by adapting the dedicated software for the LA, given the lack of dedicated RA strain algorithms. An illustrative figure demonstrating the strain curves and the measurement of dispersion has been added to the Supplementary Materials (Supplementary Figure S1).

### 2.3 Electrophysiological study and catheter ablation

For patients with AF, the aim of CA was to obtain electrical isolation of pulmonary veins (PVI), with adjunctive left atrial linear or focal ablation in selected cases, as described elsewhere (4). PVI was obtained using two techniques, involving either radiofrequency or cryothermal energy. Based on operator preference, the cryoballoon single-shot ablation approach to AF has been used for patients with paroxysmal atrial fibrillation (PAF). In particular, in patients with persistent AF (PeAF) for whom restoration of SR was not obtained after PVI, linear ablation at the LA “roof” (between the superior aspects of the left and right upper PVI lesions), posterior wall isolation (linear ablation of the LA roof joining the superior PVs and the LA floor joining the inferior PVs), and/or linear ablation of the lateral mitral isthmus were performed according to the judgement of electrophysiologist operator. Mapping and pacing maneuvers to assess line completeness were performed after ablation. When atrial flutter/tachycardia was established, activation mapping and ablation of the critical isthmus were obtained. Prior to performing the ablation, three-dimensional (3D) electroanatomic mapping (EAM) of the LA was obtained with the help of navigation systems (CARTO, Biosense Webster, Diamond Bar, CA, USA; RHYTHMIA HDx, Boston Scientific, MA, USA). EAM was performed with a multipolar catheter (Pentaray; IntellaMap Orion High Resolution) to obtain high-resolution electrical signals. A low-voltage area was defined as the total area of bipolar peak-to-peak voltage <0.05 mV for patients in SR and <0.24 mV in AF patients.

Furthermore, volume and total LA surface mapped data were collected.

### 2.4 Follow-up

After the CA procedure, all the patients were evaluated at the outpatient clinic on a regular basis. A surface electrocardiogram was acquired at every follow-up visit, and 24-h Holter monitoring was performed at 3- to 6-month intervals. Recurrence was defined as the detection of AF lasting more than 30 s after the blanking period based on the symptoms or ECGs performed at every visit to the outpatient clinic.

An echocardiographic re-evaluation, including 2D STE, was obtained at follow-up at least 3 months after CA.

### 2.5 Statistical analysis

Data are reported as mean ± standard deviation or median and range for continuous variables and number of subjects (%) for categorical variables, unless stated otherwise. Differences between means/medians were evaluated using the unpaired Student *t*-test/Mann–Whitney *U* test. Differences in dichotomous variables were assessed by using Fisher’s exact test. Pearson's correlation coefficient was calculated to correlate linear variables. The paired samples *t*-test was used to assess changes in LA and RA echocardiographic parameters between basal and follow-up values. Cox regression analysis was used to identify variables that were independently predictive of AF recurrence, including in multivariate model variables with a statistical *p*-value < 0.10; in the case of multiple variables with statistical *p* < 0.10 in the univariable analysis referring to the same clinical parameter, and therefore subject to collinearity (e.g., RAEF and pRASr), in light of the limited sample size and number of recurrence events, to build the multivariable analysis model, the most statistically significant or clinically relevant variable was included. A *p*-value <0.05 was considered statistically significant. Statistical analysis was performed using SPSS software v.22.0 (IBM, Armonk, NY, USA).

## 3 Results

### 3.1 General findings in patients with AF and controls

In total, 56 consecutive patients in the AF group and 32 subjects in the control group were enrolled.

The main comparisons between the two groups are shown in Table 1.

**Table 1:** Clinical, ECG, and STE findings in the AF group vs. the control group.

	AF group	Control group	*p*
	(*n* = 56)	(*n* = 32)	
Clinical characteristics			
Age, years	64 ± 10	49 ± 17	**<0**.**001**
Sex (male)	35 (62)	17 (53)	0.390
BMI, kg/m^2^	27 ± 4	24 ± 3	**0**.**003**
Hypertension	39 (69)	8 (25)	**0**.**001**
Diabetes	6 (11)	1 (3)	0.415
Smokers	19 (34)	10 (31)	0.713
Dyslipidemia	21 (37)	10 (31)	0.550
IHD	7 (13)	2 (6)	0.478
CHA_2_DS_2_-VASc score	2.5 ± 1.7	1.2 ± 1.2	**0**.**014**
LVEF, %	56 ± 10	59 ± 8	0.201
Anti-arrhythmics	39 (69)	3 (9)	**0**.**001**
Amiodarone	12 (21)	0 (0)	**0**.**003**
Beta-blockers	40 (71)	10 (31)	**<0**.**001**
ECG parameters			
Pmax, ms	131 ± 16.2	115 ± 16.1	**0**.**001**
Pmin, ms	91.7 ± 13.9	87.7 ± 17.0	0.266
Pd, ms	40.1 ± 13.3	27.7 ± 15.8	**<0**.**001**
*P*-wave A, mV	0.14 ± 0.05	0.16 ± 0.06	0.117
PTFV1, mV × ms	4.9 ± 3.0	3.1 ± 2.6	**0**.**011**
Interatrial block	20 (35)	12 (37)	1
QRSd, ms	106 ± 13.4	96 ± 24	**0**.**034**
STE parameters			
Left atrium			
LAVi, ml/m^2^	33.4 ± 16.7	22.4 ± 11.1	**0**.**001**
LAEF, %	44.8 ± 17.6	64.5 ± 10.5	**0**.**001**
pLASr, %	17.3 ± 10.2	29.0 ± 8.4	**0**.**001**
SD-regional-LA-TTP-N	0.059 ± 0.033	0.044 ± 0.025	**0**.**037**
Right atrium			
RAVi, ml/m^2^	24.4 ± 11.7	18.8 ± 10.9	**0**.**030**
RAEF, %	47.2 ± 15.7	58.5 ± 11.2	**<0**.**001**
pRASr, %	22.1 ± 12.6	29.8 ± 12.7	**0**.**009**
SD-regional-RA-TTP-N	0.048 ± 0.015	0.038 ± 0.009	**0**.**02**

Bolded *p*-values indicate statistical significance (*p* < 0.05). For further details see Methods section.

IHD, ischaemic heart disease.

The patients with AF were older, had a higher cardiovascular risk profile, and had worse *P*-wave ECG indexes compared to the control group.

The patients with AF had larger LA indexed volumes and worse LA mechanical two-dimensional STE parameters compared to the controls. In particular, LAEF, LA global strain in the reservoir phase, and the dispersion of LA regional time-to-peak of strain were found to be impaired in the AF group compared to the control participants.

Interestingly, the dimensional and functional parameters of the RA were also worse in patients with AF history compared to the control subjects. Indeed, RAEF, RA global strain in the reservoir phase, and the dispersion of RA regional time-to-peak of strain were found to be impaired in the AF group compared to the control participants.

To verify whether the clinical differences between the control group and the AF group could independently influence strain parameters and therefore be a potential confounding factor, a multivariable analysis of the association of each single strain parameter with the AF group, corrected for cardiovascular risk profile and medical therapy, was performed (Supplementary Table S1); the main differences in the LA and RA STE parameters between the AF patients and controls remained independent from the cardiovascular risk profile (including age) in the multivariable analysis, as assessed by CHA2DS2-VASc Score.

### 3.2 Comparison between AF patients: paroxysmal vs. persistent

#### 3.2.1 Clinical and STE features

In the AF group, 40 patients had PAF (71%), while 16 patients (29%) had PeAF.

The main comparisons between these two subgroups are shown in Table 2.

**Table 2:** Clinical, ECG, STE, and EAM findings in the PAF vs. PeAF patients.

	PAF group	PeAF group	*p*
	(*n* = 40)	(*n* = 16)	
Clinical characteristics			
Age, years	64.2 ± 11.1	64.5 ± 10.5	0.924
Sex (male)	24 (60)	11 (69)	0.761
BMI	27.2 ± 4.1	27.5 ± 4.0	0.813
Hypertension	25 (63)	14 (88)	0.107
Diabetes	3 (8)	3 (19)	0.331
Smokers	14 (35)	5 (31)	0.772
Dyslipidemia	16 (40)	5 (31)	0.761
IHD	3 (8)	4 (25)	0.094
CHA_2_DS_2_-VASc score	2.4 ± 1.7	2.9 ± 1.7	0.311
LVEF,%	58 ± 9.1	50 ± 10	**0**.**012**
PAPs, mmHg	28 ± 5	29 ± 5	0.839
Anti-arrhythmics	27 (68)	12 (75)	0.751
Amiodarone	10 (25)	2 (13)	0.475
Beta-blockers	26 (65)	14 (88)	0.114
ECG parameters			
Pmax, ms	132 ± 13	130 ± 21	0.787
Pmin, ms	93 ± 13	88 ± 15	0.299
Pd, ms	39 ± 10	42 ± 19	0.452
*P*-wave A, mV	0.14 ± 0.4	0.12 ± 0.5	0.216
PTFV1, ms × mV	5.1 ± 3.3	4.2 ± 2.2	0.321
Interatrial block	13 (36)	6 (40)	1
QRSd, ms	106 ± 12	105 ± 15	0.877
STE parameters			
Left atrium			
LAVi, ml/m^2^	32.3 ± 16.3	36.4 ± 17.8	0.398
LAEF, %	48.8 ± 17.2	35.3 ± 15.0	**0**.**009**
pLASr, %	18.5 ± 10.1	14.4 ± 10.4	0.188
SD-regional-LA-TTP-N	0.052 ± 0.032	0.074 ± 0.030	**0**.**027**
Right atrium			
RAVi, ml/m^2^	22.1 ± 11.6	30.2 ± 10.1	**0**.**018**
RAEF, %	48.4 ± 15.9	44.4 ± 15.4	0.403
pRASr, %	24.8 ± 12.1	15.9 ± 11.7	**0**.**017**
SD-regional-RA-TTP-N	0.043 ± 0.023	0.062 ± 0.030	**0**.**016**
EAM findings			
LA mapped area, cm^2^	174.0 ± 35.6	216.5 ± 44.8	**0**.**002**
Low-voltage LA area, cm^2^	2.2 ± 4.4	6.2 ± 12	0.089

Bolded *p*-values indicate statistical significance (*p* < 0.05). For further details see Methods section.

PAPs, pulmonary artery pressure systolic.

No significant differences were found with regard to clinical and ECG findings, except for left ventricle ejection fraction (LVEF) (lower in the PeAF group, *p* = 0.012).

Compared to the patients with PAF, the patients with PeAF had significantly lower LAEF (*p* = 0.009), with lower, but not statistically significant, pLASr values (*p* = 0.188), while no differences were found with regard to LA dimension.

Conversely, both RA indexes of volume (i.e., RAvolume) and function (i.e., global longitudinal strain in the reservoir phase and time to peak of regional strain variability) were significantly impaired in the patients with PeAF compared to the patients with PAF.

#### 3.2.2 Electrophysiological results

In the EAM, the global LA mapped area was larger in the patients with PeAF compared to those with PAF (*p* = 0.002). Moreover, the patients with PeAF had larger LA abnormal low-voltage areas compared to those with PAF, with a trend toward statistical significance (2.2 ± 4.4 vs. 6.2 ± 12, *p* = 0.089).

#### 3.2.3 Relationship between LA and RA strain parameters

A significant correlation was found between LA global strain during the reservoir phase with LA volume and LA emptying fraction, but also with *P-*wave indexes and abnormal electroanatomical area extension in the EAM.

Furthermore, a significant correlation was also found between pLASr and all dimensional and mechanical RA STE parameters (i.e., pRASr, RAEF, and RA variability of regional strain TTP).

Interestingly, a significant correlation was also found between RA function, as assessed by global strain during the reservoir phase, and all LA dimensional, mechanical, and electrical indexes (Supplementary Table S2).

### 3.3 Follow-up

#### 3.3.1 AF recurrence

Of the 56 AF patients, 46 had a complete follow-up.

Of 46 patients, 9 (20%) had AF recurrence, while the other 37 (80%) patients remained in SR. The median follow-up was 7 ± 3 months, while the median time to AF recurrence was 4.7 ± 1.3 months.

In the univariable analysis, patients with AF recurrence were older (68.0 ± 8.0 vs. 60.0 ± 12.0, *p* = 0.060) and had a higher CHA2DS2-VASc Score (4.0 ± 2.2 vs. 2.2 ± 1.5, *p* = 0.010) compared to patients in SR, while there were no differences regarding gender, anti-arrhythmia drug use, and LVEF and *P*-wave indexes. With regard to STE assessment, LA strain parameters did not differ between patients with and without AF recurrence, while RAEF and global pRASr were significantly worse in patients with AF recurrence at follow-up (*p* = 0.007 and *p* = 0.035, respectively). Similarly, a higher extension of LA low-voltage was found in the AF recurrence group compared to the SR group (Table 3).

**Table 3:** Comparison between patients with AF recurrence and those in sinus rhythm at follow-up.

	AF recurrence	Sinus rhythm	*p*
	(*n* = 9)	(*n* = 37)	
Clinical characteristics			
Age, years	68 ± 8	60 ± 12	0.060
Sex (male)	3 (33)	25 (67)	0.124
IHD	2 (22)	4 (10)	0.581
CHA_2_DS_2_-VASc score	4.0 ± 2.2	2.2 ± 1.5	**0**.**010**
LVEF, %	56 ± 9	56 ± 9	0.839
PAPs, mmHg	27 ± 3	29 ± 5	0.425
Anti-arrhythmics	6 (67)	25 (67)	1
Amiodarone	2 (22)	6 (16)	0.645
Beta-blockers	8 (88)	22 (60)	0.132
ECG parameters			
Pmax, ms	132 ± 22	134 ± 14	0.715
Pd, ms	45 ± 20	40 ± 12	0.372
PTFV1, mV × ms	5.5 ± 2.3	4.7 ± 3.2	0.496
QRSd, ms	105 ± 26	105 ± 13	0.960
STE parameters			
Left atrium			
LAVi, ml/m^2^	41.2.0 ± 17.8	31.3 ± 14.3	0.089
LAEF, %	38.2 ± 22.1	48.1 ± 17.3	0.154
pLASr, %	14.4 ± 14.2	18.2 ± 10.0	0.359
SD-regional-LA-TTP-N	0.050 ± 0.033	0.062 ± 0.036	0.380
Right atrium			
RAVi, ml/m^2^	25.4 ± 8.8	24.7 ± 11.2	0.876
RAEF, %	34.4 ± 17.9	50.8 ± 15.2	**0**.**009**
pRASr, %	14.1 ± 11.7	24.6 ± 13.5	**0**.**039**
SD-regional-RA-TTP-N	0.054 ± 0.031	0.032 ± 0.010	**0**.**002**
EAM findings			
Low-voltage LA area, cm^2^	10.3 ± 15.5	1.4 ± 3.9	**0**.**003**

Bolded *p*-values indicate statistical significance (*p* < 0.05). For further details see Methods section.

In the Cox regression multivariable analysis, the only parameter independently associated with AF recurrence was the CHA2DS2-VASc Score (HR 1.471, 95% CI 1.003–2.158, *p* = 0.048) (Table 4).

**Table 4:** Multivariable analysis of main predictors of AF recurrence, after a “blanking period” of 3 months.

	Multivariable analysis	
	HR (95% CI)	*p*
CHA_2_DS_2_-VASc score	1.471 (1.003–2.158)	**0.048**
RAEF, %	0.43 (0.003–70.210)	0.746
Low-voltage LA area, cm^2^	0.994 (0.936–1.056)	0.848
LAVi, ml/m^2^	1.027 (0.973–1.085)	0.331

Time to recurrence: 4.7 ± 1.3 months.

Bolded *p*-values indicate statistical significance (*p* < 0.05). For further details see Methods section.

#### 3.3.2 STE changes in patients with AF post-ablation

No significant changes were observed from baseline to follow-up in patients with AF recurrence regarding LA and RA strain parameters, while an increase with a trend to significance of LA contraction (LAEF from 64.5 ± 10.5–60 ± 11, *p* = 0.066) and a significant reduction of RA volume (RAVi from 24.3 ± 11.4–22 ± 11, *p* = 0.046) were found in patients who remained in SR (Supplementary Table S3).

## 4 Discussion

The present study adds to the growing literature on the role of RA in AF by investigating (1) the role of RA remodeling in patients undergoing CA for AF, and (2) the relationship between the electroanatomical atrial substrate and function through strain analysis.

The following main findings emerge from our study: (1) the patients with AF had not only worse left atrial, but also right atrial strain indexes compared to the controls; (2) the patients with PeAF showed impaired LA strain values, but even worse RA strain indexes, compared to the patients with PAF; (3) RA function, as studied with STE, showed a significant relationship with both LA strain and volume indexes and with abnormal LA electroanatomical substrate; (4) among the STE parameters, RA, but not LA, parameters were found to be significantly reduced in patients with AF recurrence post-ablation compared to patients who remained in SR, while the only independent clinical predictor of AF recurrence remained cardiovascular risk profile.

STE of the LA has permitted researchers to analyze the atrial remodeling mechanisms underlying AF (3, 18, 22, 23). Conversely, RA is often poorly considered, possibly due to the reduced accuracy in its instrumental assessment (19) and the traditional “cornerstone” of PV ablation in LA (4).

Most studies have analyzed the relationship between RA structural and electrical remodeling and the incidence of AF by focusing on specific, predominantly comorbid patient settings (13, 16, 24, 25). Notably, we investigated a sample of patients with AF without relevant pulmonary and valvular pathologies underlying RA remodeling that could link it with AF.

In the present study, we found that patients with AF had not only worse left but also right atrial strain indices than the controls. Thus, the changes underlying AF may affect both atria, and the “holistic” concept of “bi-atrial cardiomyopathy,” sustained by previous studies (3, 26), should be considered to underlie the pathophysiological nature of AF.

Interestingly, we found that the patients with PeAF showed even worse RA strain indexes compared to the patients with PAF, although with only slightly impaired LA strain indexes. Thus, when comparing more advanced forms with early forms of AF, while differences in LA alterations may be less evident, those of the RA become the most discriminating element, demonstrating earlier and more extensive remodeling. Moreover, impaired RA strain parameters were significantly correlated with greater electroanatomical complexity of the LA, as expressed by greater potential abnormal areas. Therefore, the dated notion of “AF begets AF” (27) should be reshaped in light of the “dual-chamber attribute” of AF, as the degree of bi-atrial alteration worsens from PAF to PeAF. Previous studies, albeit with low patient sample numbers, highlight interesting data about electrophysiology, bi-atrial initiation, and maintenance of AF (12, 16, 24, 28).

In our study, RA parameters were found to be significantly impaired in the patients who had AF recurrence post-ablation, compared to the patients who persisted in SR. Furthermore, the extent of low-voltage areas of the left atrium was larger, while the LA strain parameters did not differ significantly between the two groups. At baseline, we found a greater extension of the LA low-voltage area in patients with persistent vs. paroxysmal atrial fibrillation (Table 2), with a statistically significant inverse relationship between the extension of low voltages in the electroanatomical map and the strain values in the STE analysis (Supplementary Table S2). At follow-up, the subgroup of patients with post-ablation AF recurrence showed a larger extension of the low-voltage areas compared to the patients who persisted in SR, with LA strain values that did not differ significantly, although they were worse. The observed discrepancy between LA low-voltage area extension and LA strain parameters in patients with AF recurrence vs. those who remained in SR is noteworthy. While RA strain may serve as an early marker of atrial dysfunction, the lack of close correlation between LA strain and low-voltage area extension could reflect differences in the underlying pathophysiology they capture. LA strain primarily assesses mechanical deformation, whereas low-voltage areas indicate electrical remodeling and interstitial fibrosis, which may not always translate into immediate functional impairment (e.g., an incomplete parietal extension of tissue abnormalities may affect endocardial voltage but not necessarily mechanical function). Further studies are needed to elucidate these complex interactions.

Thus, a more advanced cardiomyopathy should be linked to the reduced effectiveness of AF ablation. An accurate RA strain study could allow an earlier screening of patients at risk of more complex arrhythmias and susceptible to AF recurrence at follow-up. Nonetheless, there is substantial discussion on the reference ranges for RA strain parameters, which has limited clinical practice so far (14, 29, 30).

Previously, other studies have substantially underlined the reduced effectiveness of AF ablation in patients affected by bi-atrial cardiomyopathy (31–33). In our study, the patients with AF recurrences had worse RA strain values and greater dispersion of contraction, while showing similar RA dimensions, compared to the patients in SR. In particular, RA dispersion served as a “marker” of atrial electromechanical dyssynchrony, which is closely linked to atrial remodeling in AF. The increased heterogeneity in RA strain timing suggests underlying fibrosis and structural remodeling, which are common in AF and contribute to conduction slowing and local activation delays. Thus, a greater RA dispersion may reflect atrial electrical instability, which could predispose patients to AF recurrence after catheter ablation. This is consistent with prior studies demonstrating the role of atrial dyssynchrony in maintaining AF substrates (11, 21, 28).

Hence, RA strain parameters may be a more sensitive tool, detecting subtle RA contractile stunning and patchy myocardial modifications that would otherwise not be identified with standard evaluations. Interestingly, a recent study showed that RA fibrosis, assessed by cardiac magnetic resonance, was not predictive of AF recurrence after AF ablation (34). It is noteworthy that it did not provide analyses of data findings in different subtypes of AF, but showed a strong correlation of RA and LA remodeling parameters in the whole population.

Moreover, we sought to determine the correlation between STE RA findings and the LA electrophysiological substrate. Not only impaired left but also RA strain parameters were significantly correlated with low-voltage area extension, confirming data from the literature that demonstrate LAS as an indicator of AF progression (35, 36), paving the way for the RAS to play an important role in this field.

It should be emphasized that RA strain parameters did not remain independent predictors in the multivariable models, where only the CHA2DS2-VASc score was predictive. However, possible explanations for this result include the limited sample size and number of recurrence events, potential collinearity with clinical variables, and limitations in capturing the complexity of the arrhythmic substrate solely through RA strain.

These findings require future research in larger cohorts.

Overall, the results of the present study suggest that RA strain analysis may be a valuable tool to identify patients with more advanced atrial cardiomyopathy and at higher risk of AF recurrence after PVI ablation and who may therefore benefit from an extended ablation strategy beyond PVI. In particular, individuals exhibiting high RA strain heterogeneity may be more suitable for targeted ablation of non-PV triggers, such as superior vena cava isolation, crista terminalis ablation, or coronary sinus modification. In addition, a “bi-atrial substrate modification approach,” incorporating the ablation of low-voltage areas or fractionated electrograms, may be warranted in selected cases (4, 17). This perspective aligns with emerging evidence indicating that patients with significant RA involvement may experience improved outcomes when adjunctive non-PV ablation strategies are implemented.

Interestingly, we also found that only the patients who remained in SR after CA at follow-up showed an improvement in RA volume (i.e., reverse atrial remodeling), unlike the patients with AF recurrence.

The most significant limitations of our study were a relatively small sample size and brief follow-up, so our findings need to be validated in larger populations. Furthermore, substrate mapping of RA, not performed in our group of patients, would have been useful to better investigate the relationship between mechanical and electrical function in this cardiac chamber. In addition, a comparison of echocardiographic measurements of RA performance with other imaging modalities could have provided insightful data on atrial remodeling status. Moreover, currently available STE software was developed for assessment of LA function and its use in the RA has not yet been fully validated; it must be taken into account that its extension to the RA is limited by several factors (anatomical issues, variability in acoustic windows, and lack of standardized algorithms) (19), and the results could have been affected by differences in software platforms and post-processing techniques (vendor-related variability).

Finally, a few echocardiographic studies were conducted on AF rhythm (patients with PeAF): even if some values were corrected for cycle length, this circumstance may have altered appraisals, besides reducing the number of achievable parameters.

## 5 Conclusion

Two-dimensional STE is a useful and accessible method of assessment for both RA and LA function in patients with AF. Our study suggests that a careful evaluation of right atrial strain parameters could provide information on bi-atrial disease progression and different possible clinical outcomes. In particular, RA reservoir strain function was progressively impaired in the more severe subtypes of AF, displaying a strong correlation with both LA mechanical and functional parameters. Moreover, some electrophysiological features, known to define more pathological substrates, showed a significant linear relationship with STE characteristics of the RA. In the cutting-edge debate on “non-PV triggers” in CA, these data could contribute to a better strategy definition.

Further studies are required to confirm these results.

## Data Availability

The datasets presented in this study can be found in online repositories. The names of the repository/repositories and accession number(s) can be found in the article/Supplementary Material.

## References

[1] Staerk L Sherer JA Ko D Benjamin EJ Helm RH. Atrial fibrillation: epidemiology, pathophysiology, and clinical outcomes. Circ Res. (2017) 120(9):1501. 10.1161/CIRCRESAHA.117.309732 28450367 PMC5500874

[2] Patel DA Lavie CJ Milani RV Shah S Gilliland Y. Clinical implications of left atrial enlargement: a review. Ochsner J. (2009) 9(4):191. 21603443 PMC3096293

[3] Goette A Kalman JM Aguinaga L Akar J Cabrera JA Chen SA EHRA/HRS/APHRS/SOLAECE expert consensus on atrial cardiomyopathies: definition, characterization, and clinical implication. Hear Rhythm. (2017) 14(1):e3. 10.1016/j.hrthm.2016.05.028 PMC5548137 27320515

[4] Calkins H Hindricks G Cappato R Kim YH Saad EB Aguinaga L 2017 HRS/EHRA/ECAS/APHRS/SOLAECE expert consensus statement on catheter and surgical ablation of atrial fibrillation. Europace. (2017) 20(1):e1–160. 10.1093/europace/eux274 PMC5834122 29016840

[5] Mujović N Marinković M Lenarczyk R Tilz R Potpara TS. Catheter ablation of atrial fibrillation: an overview for clinicians. Adv Ther. (2017) 34(8):1897. 10.1007/s12325-017-0590-z 28733782 PMC5565661

[6] Abazid RM Smettie O Romsa JG Warrington J Akincioglu C Tzemos N Atrial anatomical variations on computed tomography angiography associated with atrial fibrillation and those predicting recurrence following pulmonary vein isolation. Radiol Adv. (2024) 1(2):umae016. 10.1093/radadv/umae016 PMC12429179 41059390

[7] Tops LF Delgado V Bertini M Marsan NA Den Uijl DW Trines SAIP Left atrial strain predicts reverse remodelling after catheter ablation for atrial fibrillation. J Am Coll Cardiol. (2011) 57(3):324–31. 10.1016/j.jacc.2010.05.063 21232671

[8] Khan HR Yakupoglu HY Kralj-Hans I Haldar S Bahrami T Clague J Left atrial function predicts atrial arrhythmia recurrence following ablation of long-standing persistent atrial fibrillation. Circ Cardiovasc Imaging. (2023) 16(6):E015352. 37288553 10.1161/CIRCIMAGING.123.015352 PMC10281195

[9] Johansen MC De Vasconcellos HD Nazarian S Lima JAC Gottesman RF. The investigation of left atrial structure and stroke etiology: the i-laser study. J Am Heart Assoc. (2021) 10(2):1–10. 10.1161/JAHA.120.018766 PMC7955322 33442991

[10] Wałek P Grabowska U Cieśla E Gorczyca I Wożakowska-Kapłon B. Left atrial longitudinal strain in the contractile phase as a predictor of sinus rhythm maintenance after electrical cardioversion performed due to persistent atrial fibrillation. Kardiol Pol. (2021) 79(4):458–60. 10.33963/KP.15913 33784038

[11] Yasuda R Murata M Roberts R Tokuda H Minakata Y Suzuki K Left atrial strain is a powerful predictor of atrial fibrillation recurrence after catheter ablation: study of a heterogeneous population with sinus rhythm or atrial fibrillation. Eur Heart J Cardiovasc Imaging. (2015) 16(9):1008–14. 25750193 10.1093/ehjci/jev028

[12] Saksena S Skadsberg ND Rao HB Filipecki A. Biatrial and three-dimensional mapping of spontaneous atrial arrhythmias in patients with refractory atrial fibrillation. J Cardiovasc Electrophysiol. (2005) 16(5):494–504. 10.1111/j.1540-8167.2005.40531.x 15877620

[13] Soulat-Dufour L Lang S Ederhy S Ancedy Y Beraud AS Adavane-Scheuble S Biatrial remodelling in atrial fibrillation: a three-dimensional and strain echocardiography insight. Arch Cardiovasc Dis. (2019) 112(10):585–93. 10.1016/j.acvd.2019.06.010 31540880

[14] Lang RM Cameli M Sade LE Faletra FF Fortuni F Rossi A Imaging assessment of the right atrium: anatomy and function. Eur Hear J - Cardiovasc Imaging. (2022) 6:1–18. 10.1093/ehjci/jeac011 35079782

[15] Hindricks G Potpara T Dagres N Bax JJ Boriani G Dan GA 2020 ESC guidelines for the diagnosis and management of atrial fibrillation developed in collaboration with the European association for cardio-thoracic surgery (EACTS). Eur Heart J. (2020) 42(5):373–498. 10.1093/eurheartj/ehaa612 32860505

[16] Lim HS Denis A Middeldorp ME Lau DH Mahajan R Derval N Persistent atrial fibrillation from the onset: a specific subgroup of patients with biatrial substrate involvement and poorer clinical outcome. JACC Clin Electrophysiol. (2016) 2(2):129–39. 10.1016/j.jacep.2015.12.014 29766861

[17] Calkins H Kuck KH Cappato R Brugada J Camm AJ Chen SA 2012 HRS/EHRA/ECAS expert consensus statement on catheter and surgical ablation of atrial fibrillation: recommendations for patient selection, procedural techniques, patient management and follow-up, definitions, endpoints, and research trial design. Europace. (2012) 14(4):528–606. 10.1093/europace/eus027 22389422

[18] Lang RM Badano LP Mor-Avi V Afilalo J Armstrong A Ernande L Recommendations for cardiac chamber quantification by echocardiography in adults: an update from the American society of echocardiography and the European association of cardiovascular imaging. Eur Heart J Cardiovasc Imaging. (2015) 16(3):233–71. 10.1093/ehjci/jev014 25712077

[19] Badano LP Kolias TJ Muraru D Abraham TP Aurigemma G Edvardsen T Standardization of left atrial, right ventricular, and right atrial deformation imaging using two-dimensional speckle tracking echocardiography: a consensus document of the EACVI/ASE/industry task force to standardize deformation imaging. Eur Heart J Cardiovasc Imaging. (2018) 19(6):591–600. 10.1093/ehjci/jey042 29596561

[20] Mouselimis D Tsarouchas AS Pagourelias ED Bakogiannis C Theofilogiannakos EK Loutradis C Left atrial strain, intervendor variability, and atrial fibrillation recurrence after catheter ablation: a systematic review and meta-analysis. Hellenic J Cardiol. (2020) 61(3):154–64. 10.1016/j.hjc.2020.04.008 32325233

[21] Kawakami H Ramkumar S Nolan M Wright L Yang H Negishi K Left atrial mechanical dispersion assessed by strain echocardiography as an independent predictor of new-onset atrial fibrillation: a case-control study. J Am Soc Echocardiogr. (2019) 32(10):1268–1276.e3. 10.1016/j.echo.2019.06.002 31466848

[22] Hopman LHGA Mulder MJ van der Laan AM Demirkiran A Bhagirath P van Rossum AC Impaired left atrial reservoir and conduit strain in patients with atrial fibrillation and extensive left atrial fibrosis. J Cardiovasc Magn Reson. (2021) 23(1):131. 34758820 10.1186/s12968-021-00820-6 PMC8582184

[23] Zhong Xf Liu Ds Zheng Yq Peng Gj Sheng Yy Chen Lx Left atrial reservoir and pump function after catheter ablation with persistent atrial fibrillation: a two-dimensional speckle tracking imaging study. Acta Cardiol. (2023) 78(3):331–40. 10.1080/00015385.2022.2076308 35904446

[24] Baykaner T Clopton P Lalani GG Schricker AA Krummen DE Narayan SM. Targeted ablation at stable atrial fibrillation sources improves success over conventional ablation in high-risk patients: a substudy of the CONFIRM trial. Can J Cardiol. (2013) 29(10):1218–26. 10.1016/j.cjca.2013.07.672 23993247 PMC3787988

[25] Alenezi F Mandawat A Il’Giovine ZJ Shaw LK Siddiqui I Tapson VF Clinical utility and prognostic value of right atrial function in pulmonary hypertension. Circ Cardiovasc Imaging. (2018) 11(11):e006984. 10.1161/CIRCIMAGING.117.006984 30571314 PMC6309911

[26] Schotten U Verheule S Kirchhof P Goette A. Pathophysiological mechanisms of atrial fibrillation: a translational appraisal. Physiol Rev. (2011) 91(1):265–325. 10.1152/physrev.00031.2009 21248168

[27] Lu Z Scherlag BJ Lin J Niu G Fung KM Zhao L Atrial fibrillation begets atrial fibrillation: autonomic mechanism for atrial electrical remodelling induced by short-term rapid atrial pacing. Circ Arrhythm Electrophysiol. (2008) 1(3):184–92. 10.1161/CIRCEP.108.784272 19808412 PMC2766842

[28] Stiles MK John B Wong CX Kuklik P Brooks AG Lau DH Paroxysmal lone atrial fibrillation is associated with an abnormal atrial substrate. Characterizing the “second factor”. J Am Coll Cardiol. (2009) 53(14):1182–91. 10.1016/j.jacc.2008.11.054 19341858

[29] Gorcsan J 3rd Orihara Y. Clinical importance and current problems with right atrial strain measurements. JACC Cardiovasc Imaging. (2023) 16(3):295–7. 10.1016/j.jcmg.2023.01.010 36889848

[30] Krittanawong C Maitra NS Hassan Virk HU Farrell A Hamzeh I Arya B Normal ranges of right atrial strain: a systematic review and meta-analysis. JACC Cardiovasc Imaging. (2023) 16(3):282–94. 10.1016/j.jcmg.2022.06.022 36648033

[31] Akutsu Y Kaneko K Kodama Y Suyama J Li HL Hamazaki Y Association between left and right atrial remodelling with atrial fibrillation recurrence after pulmonary vein catheter ablation in patients with paroxysmal atrial fibrillation a pilot study. Circ Cardiovasc Imaging. (2011) 4(5):524–31. 10.1161/CIRCIMAGING.110.962761 21778328

[32] Govindan M Kiotsekoglou A Saha SK Camm AJ. Right atrial myocardial deformation by two-dimensional speckle tracking echocardiography predicts recurrence in paroxysmal atrial fibrillation. J Echocardiogr. (2017) 15(4):166–75. 10.1007/s12574-017-0341-9 28639243

[33] Shin SH Park MY Oh WJ Hong SJ Pak HN Song WH Left atrial volume is a predictor of atrial fibrillation recurrence after catheter ablation. J Am Soc Echocardiogr. (2008) 21(6):697–702. 10.1016/j.echo.2007.10.022 18187293

[34] Hopman LHGA Visch JE Bhagirath P van der Laan AM Mulder MJ Razeghi O Right atrial function and fibrosis in relation to successful atrial fibrillation ablation. Eur Heart J Cardiovasc Imaging. (2023) 24(3):336–45. 10.1093/ehjci/jeac152 35921538 PMC9936834

[35] Verma A Wazni OM Marrouche NF Martin DO Kilicaslan F Minor S Pre-existent left atrial scarring in patients undergoing pulmonary vein antrum isolation: an independent predictor of procedural failure. J Am Coll Cardiol. (2005) 45(2):285–92. 10.1016/j.jacc.2004.10.035 15653029

[36] John B Stiles MK Kuklik P Chandy ST Young GD MacKenzie L Electrical remodelling of the left and right atria due to rheumatic mitral stenosis. Eur Heart J. (2008) 29(18):2234–43. 10.1093/eurheartj/ehn329 18621772

