# Supplementary material for: Right atrial strain in atrial fibrillation: the hidden side of the moon
Source: Front Cardiovasc Med. 2025 May 30;12:1578524. doi: 10.3389/fcvm.2025.1578524 (PMC12162598; doi:10.3389/fcvm.2025.1578524)

Supplementary Material

## Supplementary Tables

**Supplementary Table 1.** Multivariable analysis for association of single strain parameters with AF group corrected for cardiovascular risk profile and medical therapy

|  |  | **Multivariable Analysis** | |
| --- | --- | --- | --- |
|  |  | **OR (95%, CI)** | ***p*** |
| CHA_2_DS_2_ VASc Score |  | 2.63 (1.21-5.74) | **0.015** |
| Beta-blockers |  | 6.21 (0.48-78) | 0.161 |
| Anti-arrhythmics |  | 58 (4-92) | **0.004** |
| LAEF, % |  | 0.001 (0.0001-0.234) | **0.036** |
| pLASr, % |  | 0.820 (0.687-0.978) | **0.027** |
| SD-regional-LA-TTP-N |  | 11 (2-137) | **0.016** |
| pRASr, % |  | 0.94 (0.88-0.99) | **0.048** |
| RAEF, % |  | 0.006 (0.001-0.57) | **0.028** |

**Supplementary Table 2.** Significant correlations between ECG, STE and EP variables in the whole population.

|  | **Pearson correlation (r)*** | ***p*** |
| --- | --- | --- |
| ***pLASr***^+^ |  |  |
| LAVi, mL/m2 | -0.688 | **<0.001** |
| LAEF, % | 0.822 | **<0.001** |
| SD-regional-LA-TTP-N, % | -0.311 | **0.005** |
| RAVi, mL/m2 | -0.295 | **0.007** |
| RAEF, % | 0.461 | **<0.001** |
| pRASr, % | 0.594 | **<0.001** |
| SD-regional-RA-TTP-N, % | -0.355 | **0.001** |
| Pmax, ms | -0.344 | **0.002** |
| Low-Voltage LA area, cm2 | -0.274 | **0.039** |
| ***pRASr***^+^ |  |  |
| LAVi, mL/m2 | -0.250 | **0.022** |
| LAEF, % | 0.565 | **<0.001** |
| pLASr, % | 0.594 | **<0.001** |
| SD-regional-LA-TTP-N | -0252 | **0.024** |
| RAVi, mL/m2 | -0.484 | **<0.001** |
| RAEF, % | 0.672 | **<0.001** |
| SD-regional-LA-TTP-N | -0.451 | **<0.001** |
| Pmax, ms | -0.353 | **0.002** |
| Low-Voltage LA area,cm2 | -0.316 | **0.018** |

^+For abbreviations see text.^

**Supplementary Table 3.** STE parameters changes in AF Group vs Sinus Rhythm Group after ablation.

|  | **AF Recurrence (n=9)** | | | **Sinus Rhythm (n=37)** | | | |
| --- | --- | --- | --- | --- | --- | --- | --- |
|  | **Baseline** | **Follow-up** | ***p*** | | **Baseline** | **Follow-up** | ***p*** |
| pLASr, % | 17.4±15.0 | 20.8±10.3 | 0.566 | | 19.5±10.6 | 22.2±10.8 | 0.322 |
| LAEF, % | 43.5±25.6 | 47.5±15.2 | 0.723 | | 51.6±17.9 | 60.9±11.2 | **0.066** |
| SD-regional-LA-TTP-N | 0.063±0.023 | 0.060±0.036 | 0.878 | | 0.060±0.033 | 0.039±0.017 | **0.017** |
| pRASr, % | 16.0±12.6 | 21.1±9.7 | 0.318 | | 26.2±14.4 | 24.7±9.4 | 0.650 |
| RAEF, % | 36.3±18.4 | 40.4±13.1 | 0.629 | | 50.5±14.6 | 50.7±16.6 | 0.966 |
| SD-regional-RA-TTP-N | 0.029±0.007 | 0.060±0.034 | 0.088 | | 0.052±0.030 | 0.050±0.027 | 0.759 |
| LAVi, mL/m2 | 38.2±19.3 | 36.2±16.9 | 0.785 | | 28.5±12.3 | 32.5±14.1 | 0.170 |
| RAVi, mL/m2 | 25.3±8.7 | 26.0±7.8 | 0.878 | | 27.3±10.3 | 22.1±11.9 | **0.046** |

**1.2 Supplementary Figures**

**Supplementary Figure 1.** Measurements of atrial strain. Left panel (1): LA strain components. Central panel (2): measurements of LA dispersion and representative cases in patients with (2A) and without (2B) AF (B). Arrows indicate time-to-peak (TTP, ms) values, defined as the time interval from the end diastole (the Rwave on the electrocardiogram trace) to the peak of positive deformation, in each LA segment (septum, lateral and roof wall). SD-regional-LA-TTP was calculated as the SD of TTP and expressed as a percentage of the R-R interval. Right panel (3): an equivalent methodology to that of the LA was used for RA.


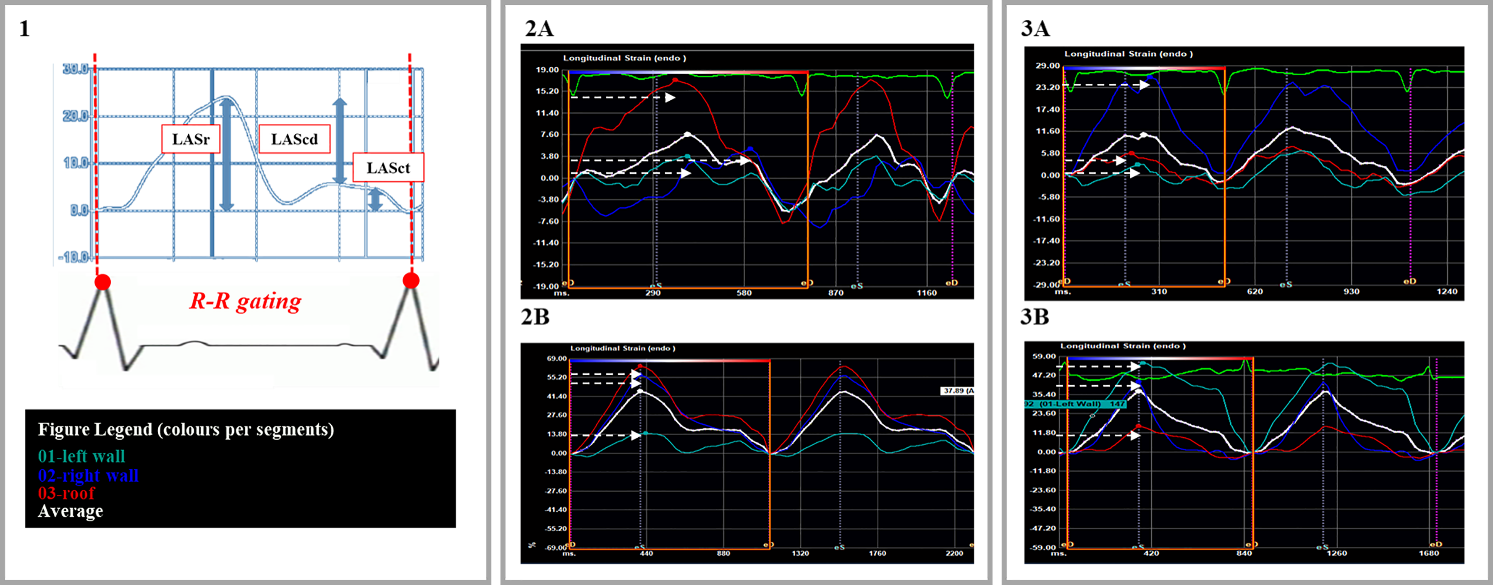

Supplement: Supplementary file 1 [file Supplementaryfile1.docx]
